# Wave–particle interaction effects in the Van Allen belts

**DOI:** 10.1186/s40623-021-01508-y

**Published:** 2021-10-19

**Authors:** Daniel N. Baker

**Affiliations:** Laboratory for Atmospheric and Space Physics, University of Colorado Boulder, 3665 Discovery Drive, 600 UCB, Boulder, CO 80303 USA

**Keywords:** Plasma waves, Energetic particles, Radiation belts, Acceleration

## Abstract

Discovering such structures as the third radiation belt (or “storage ring”) has been a major observational achievement of the NASA Radiation Belt Storm Probes program (renamed the “Van Allen Probes” mission in November 2012). A goal of that program was to understand more thoroughly how high-energy electrons are accelerated deep inside the radiation belts—and ultimately lost—due to various wave–particle interactions. Van Allen Probes studies have demonstrated that electrons ranging up to 10 megaelectron volts (MeV) or more can be produced over broad regions of the outer Van Allen zone on timescales as short as a few minutes. The key to such rapid acceleration is the interaction of “seed” populations of ~ 10–200 keV electrons (and subsequently higher energies) with electromagnetic waves in the lower band (whistler-mode) chorus frequency range. Van Allen Probes data show that “source” electrons (in a typical energy range of one to a few tens of keV energy) produced by magnetospheric substorms play a crucial role in feeding free energy into the chorus waves in the outer zone. These chorus waves then, in turn, rapidly heat and accelerate the tens to hundreds of keV seed electrons injected by substorms to much higher energies. Hence, we often see that geomagnetic activity driven by strong solar storms (coronal mass ejections, or CMEs) commonly leads to ultra-relativistic electron production through the intermediary step of waves produced during intense magnetospheric substorms. More generally, wave–particle interactions are of fundamental importance over a broad range of energies and in virtually all regions of the magnetosphere. We provide a summary of many of the wave modes and particle interactions that have been studied in recent times.

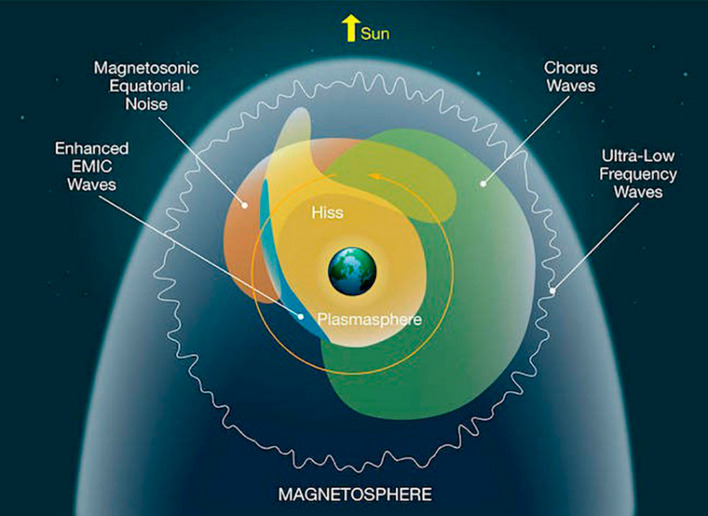

## Introduction

It is often asserted that the Earth’s near-space environment is a vast plasma physical laboratory. Key earlier papers have examined in considerable theoretical depth the principal wave modes likely affecting electron acceleration and loss in the radiation belts (Summers et al. 2007). Perhaps no spacecraft mission has more clearly or capably been able to examine experimentally the wave–particle interactions than NASA’s Radiation Belt Storm Probes (RBSP) program. The dual-satellite RBSP vehicles were fully instrumented to measure magnetic fields, electric fields, plasma electrons and ions, and a full spectrum of radiation belt energetic particles. The state-of-the-art sensors were geared toward understanding deeply how radiation belt particles were accelerated, transported, and lost from the Van Allen belt regions surrounding the Earth (Mauk et al. 2013).

The RBSP spacecraft were instrumented with identical sensors onboard the two vehicles. The satellites were launched together into a geostationary transfer orbit (GTO) on 30 August 2012. Only about 2 days after the successful launch and RBSP orbit insertion, the scientific sensors began to be switched on. Notably, the Relativistic Electron–Proton Telescopes (Baker et al. 2013a) on both the RBSP-A and RBSP-B spacecraft began acquiring data on 1 September 2012. Almost immediately, the Relativistic Electron–Proton Telescope (REPT) sensor made new discoveries about the morphology of the outer Van Allen radiation belt (Baker et al. 2013b). The detection and characterization of the so-called relativistic electron “storage ring” (or third Van Allen belt) was the beginning of a whole series of new discoveries and insights into the structure and dynamics of the radiation regions shrouding our planet. The understanding that came from the RBSP data continued at an impressive pace from the beginning of 2012 until the two RBSP spacecraft ran out of station-keeping fuel in 2019 and the mission was brought to an operational end (Baker et al. 2021).

In this paper, we will focus on wave–particle effects that were studied using the comprehensive RBSP data sets. Much of our attention will be directed toward the understanding gained about relativistic and ultra-relativistic electrons (*E* ~ 1 MeV to *E* > 10 MeV) in the outer Van Allen radiation zone. Our review will discuss extensions of earlier groundbreaking work (Meredith et al. 2003) showing the central role of chorus waves in high-energy electron production in the outer zone. We will review measurements from the REPT sensors used in conjunction with plasma, fields, and lower energetic particle sensors to portray several of the mechanisms that are at play in the Earth’s magnetosphere. In doing so, we will examine a number of wave modes and their effects.

## Interplanetary shock effects and MHD waves

Events that modulate or accelerate energetic particle fluxes in the magnetosphere on very large scales are often driven by the interplanetary shock waves. Such shocks striking the magnetosphere can produce strong transient electric field pulses that propagate rapidly through the entire magnetosphere (Wilken et al. 1982; Foster et al. 2015; Kanekal et al. 2016). Under some circumstances, an interplanetary shock hitting the magnetosphere can produce a local depletion of energetic electron fluxes, while in many other cases, there can be rapid, powerful flux enhancements extending up to multi-megavolt energies (Schiller et al. 2017). Strong magnetohydrodynamic (MHD) waves produced by shock sudden impulse impacts on the magnetosphere can produce long lasting “ringing” of broad regions of the inner magnetosphere.

**Fig. 1:**
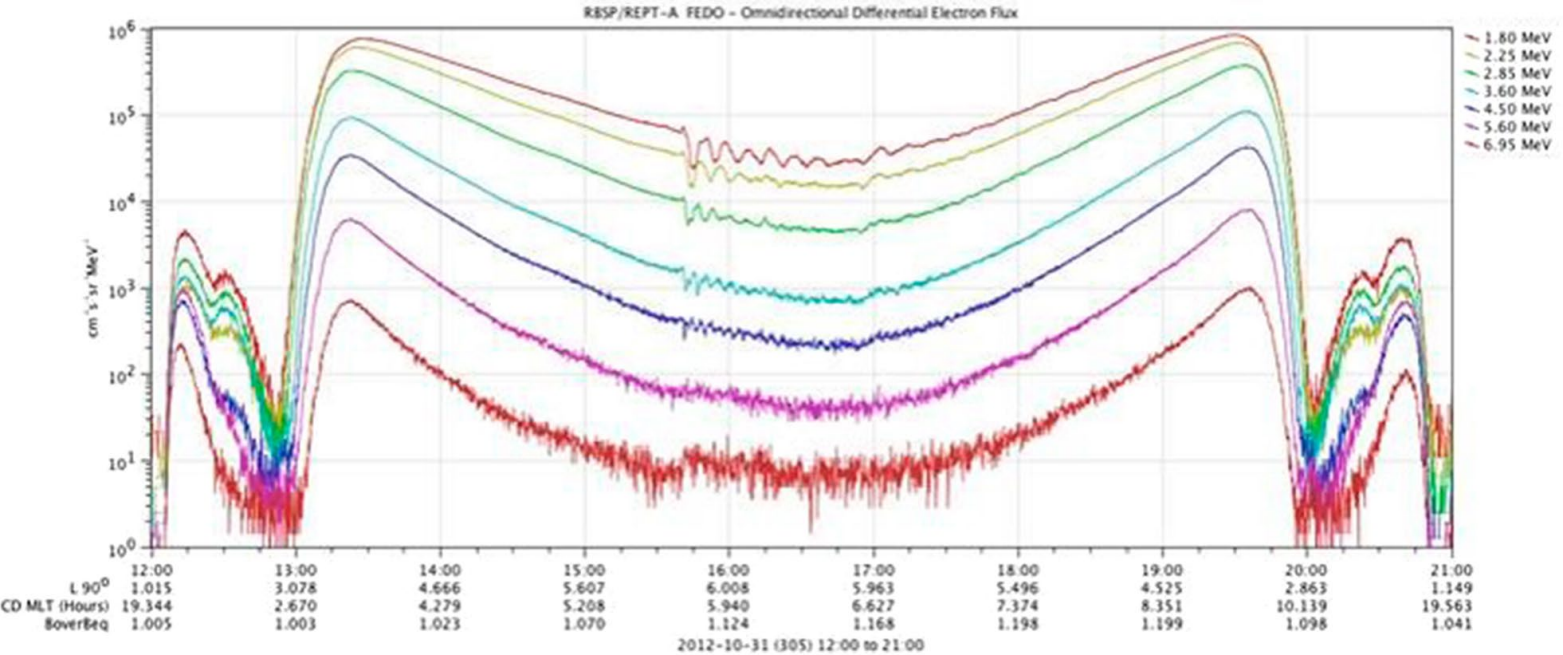
Observations from the Relativistic Electron–Proton Telescope (REPT) instrument on board the RBSP-A spacecraft for the period 1200–2100 UT on 21 October 2012. Measurements cover the electron energy range from *E* = 1.80 MeV up to *E* = 6.95 MeV as indicated by the different colored curves. A shock wave struck the Earth’s magnetosphere at ~ 1540 UT causing abrupt oscillatory flux variations as discussed in the text

Figure 1 shows an example of a strong shock wave that struck the magnetosphere at ~ 1540 UT on 31 October 2012. The RBSP-A spacecraft was near apogee (geocentric radial distance *r* ~ 5.8 Earth radii, *R*_E_) at the time of the impact and was near the dawn meridian in magnetic local time (MLT). Figure 1 shows one 9-h orbit’s worth of RBSP-A data from perigee at 1200 UT to the next perigee at 2100 UT on 31 October. The REPT data from several electron channels in the figure (ranging from *E* = 1.8 MeV to *E* = 6.95 MeV) show that the shock impact produced a clear oscillatory wave pattern in the flux time profiles extending up to at least *E* ~ 5.0 MeV. Effects were seen to even higher energies (*E* = 5.6 and *E* = 6.95 MeV) but the oscillatory behavior was masked by statistical fluctuations in the counting rates of the highest channels. The dominant effect in the electron fluxes appeared to be an initial reduction in the flux (for 1.8–4.5 MeV electrons) followed by a periodic recovery of the flux. The periodic wave modulation of fluxes lasted for well over an hour with more rapid flux oscillations (higher frequencies) at the higher energies. At the highest energy shown in Fig. 1, the flux of electrons increased quite visibly as a result of the shock impact.

More comprehensive observations of particle and field signatures for a strong shock event were presented by Foster et al. (2015). At ~ 2021 UT on 8 October 2013, a powerful IP shock struck the magnetosphere. The event was observed not only by the RBSP-A and -B spacecraft, but also by sensors on board the several THEMIS spacecraft as well (Foster et al. 2015). The RBSP-A spacecraft was deep inside the magnetosphere at *r* ~ 3 *R*_E_ at the time of the shock impact, while RBSP-B was at higher altitude near *r* ~ 4.5 *R*_E_. Both RBSP spacecraft were in the afternoon LT sector.

**Fig. 2:**
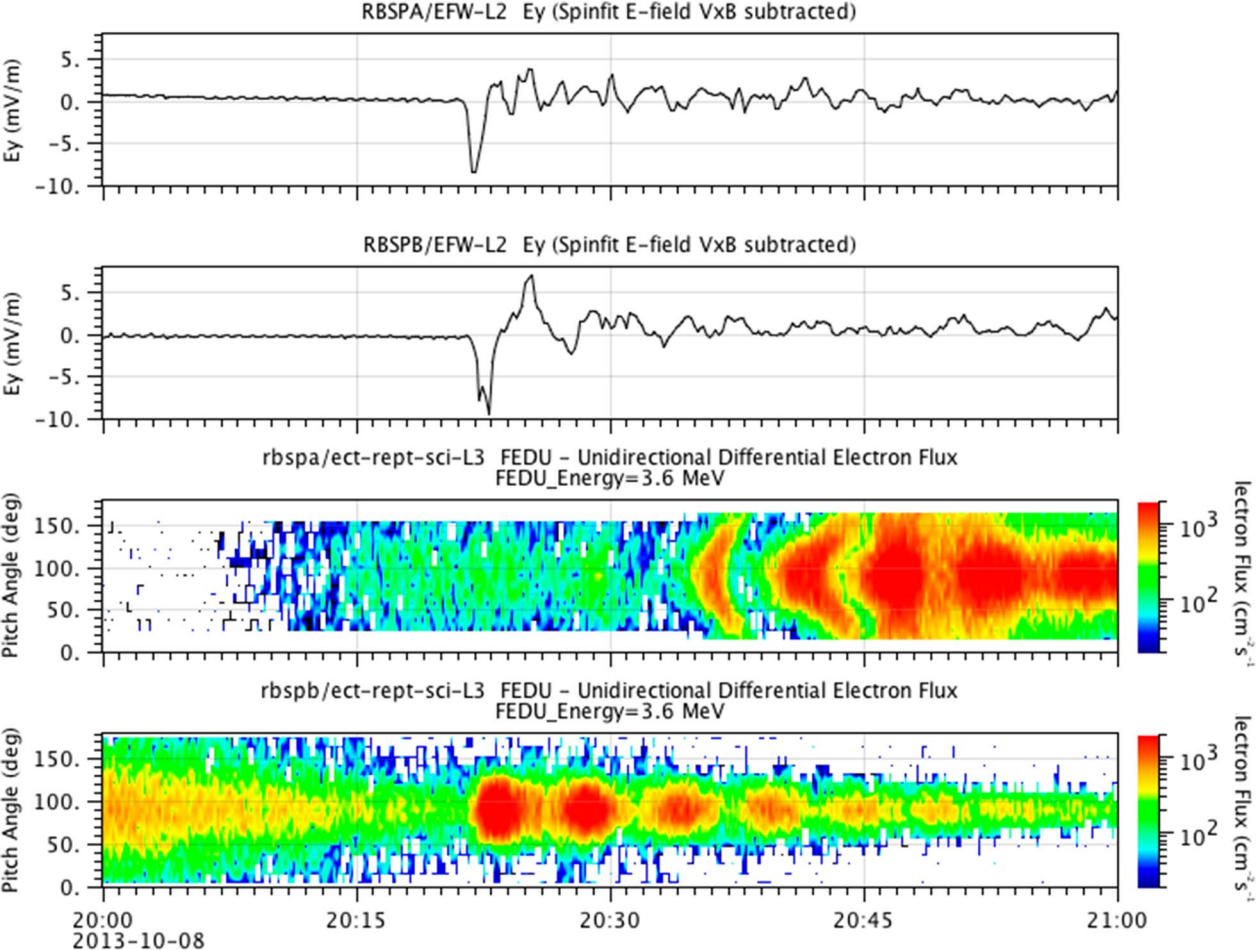
Relativistic electron flux increases in the 3.6 MeV channel from the REPT instrument on Van Allen Probes A and B is shown. (top) The shock-driven electric field at each spacecraft is included for reference. (bottom) Measurements of electron flux in the 3.6 MeV REPT channel are shown as functions of pitch angle and time from each of the two probes (From Foster et al. 2015)

Figure 2 shows in the upper two panels the azimuthal electric field profiles for RBSP-A and RBSP-B, respectively, for the 1-h period 2000–2100 UT. The large-amplitude electric field pulse resulting from the shock impact was evident at both spacecraft locations (with a very slightly earlier-in-time signal for the “A” spacecraft). The lower two panels of Fig. 2 show the pitch angle resolved energetic electron data from the identical *E* = 3.6 MeV channels of the REPT-A sensor (third panel) and the REPT-B sensor (fourth panel). Note the very prompt enhancement of 90° pitch angle electrons for REPT-B as soon as the shock wave hit the magnetosphere. There then were several subsequent “drift echo” enhancements as the initial population of electrons produced by the shock impact drifted completely around the Earth multiple times over the next 40 or so minutes (Foster et al. 2015). The REPT-A sensor also saw drift echo signatures of *E* = 3.6 MeV electrons after 2030 UT due to the shock event, but did not see the electron enhancement as promptly as the “B” spacecraft. Clearly, the strength and timing of such shock effects depends on local time and radial locations of the observing platforms. In fact, for this (and many other) IP shock events during the RBSP (or “Van Allen Probes”) era, shock impacts produced rapid flux enhancements to energies well above *E* = 6 MeV (Foster et al. 2015; Kanekal et al. 2016; Schiller et al. 2017). Clearly these types of events are global scale MHD wave-related disturbances.

## Higher frequency plasma wave phenomena

**Fig. 3:**
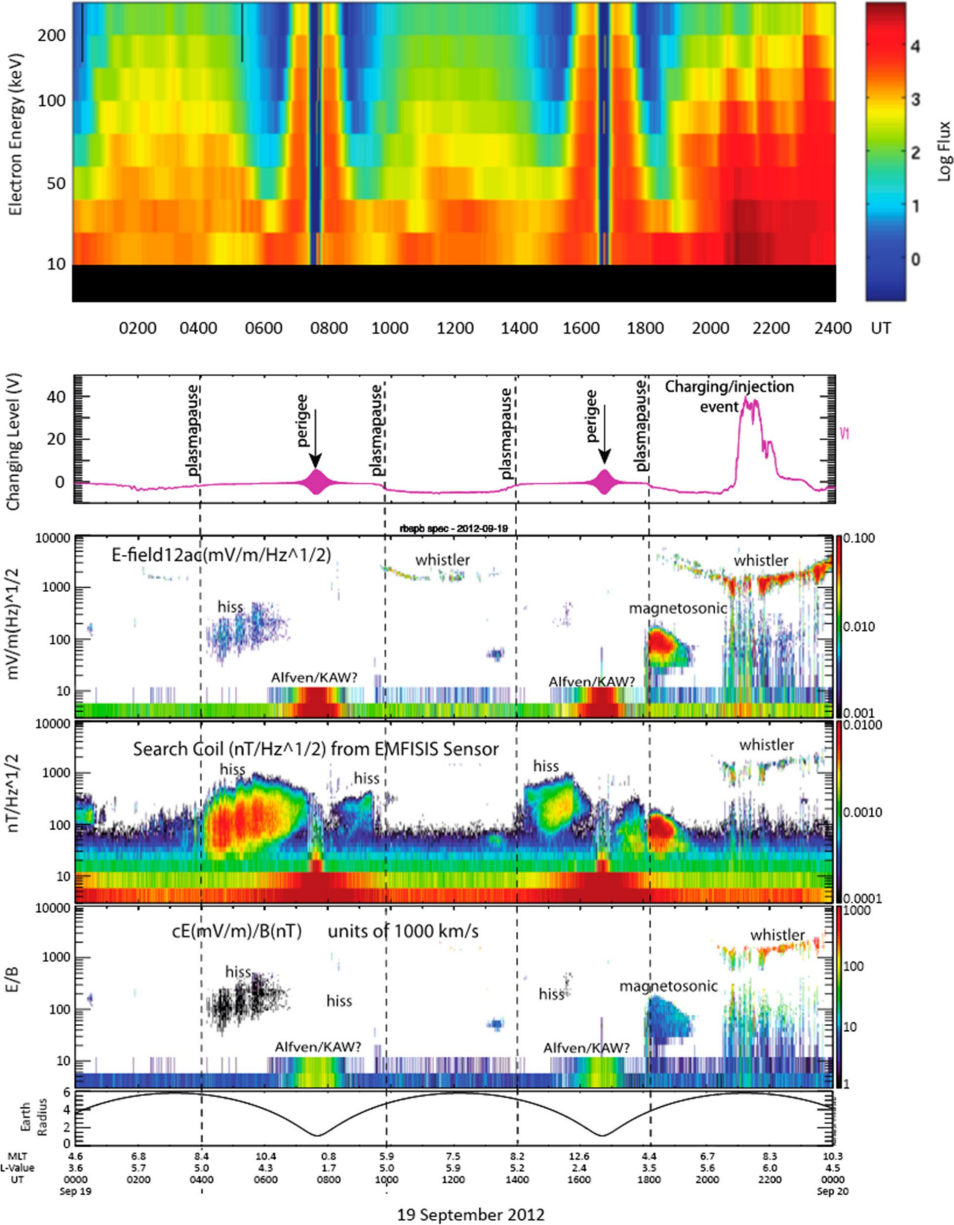
Collection of data from several instruments on board the RBSP-B spacecraft on 19 September 2012. The upper panel shows an energy-time spectrogram (color-coded) for the entire day, while the second panel shows the level of spacecraft charging inferred for the same period. Panels 3, 4, and 5 show various wave modes identified from the RBSP sensors (as described in the text). The lower panel shows the spacecraft geocentric radial distance as a function of time

The Van Allen Probes had onboard sensors capable of measuring wave phenomena across the entire spectrum of frequencies and wavelengths relevant for radiation belt particle acceleration and loss. An illustration of this is shown in Fig. 3. This portrays RBSP-B combined data for low and medium energy electrons, electric field data, and plasma wave data for a representative day (19 September 2012) shortly after the RBSP launch. Note also that the times of plasmapause crossings (as inferred from the field and plasma wave data) are also indicated by the vertical dashed lines.

The upper panel of Fig. 3 shows color-coded (log flux) energy-time intensities of *E* ~ 40 to *E* ~ 200 keV electrons for RBSP-B from the low-range head of the MagEIS instrument (Blake et al. 2013). Several magnetospheric substorm injection events were evident around times when RBSP-B was near apogee. The second panel of Fig. 3 shows the inferred spacecraft charging level over time based on Electric Field and Waves (EFW) measurements (see Wygant et al. 2013). A large charging event reaching a potential near 40 V was seen around 2000 UT associated with the strong substorm injection seen in the MagEIS data as show in the top panel.

The third panel of Fig. 3 shows wave data from a few Hertz (Hz) to $\sim$ 10 kHz. Such measurements reveal low-frequency (Alfven wave) activity as well as plasmapheric hiss waves (around 100 Hz), magnetosonic waves (just outside the plasmapause as labelled), and broadband whistler waves that are most intense during substorm injections of low and moderate energy electrons (see Panel 1). The fourth and fifth panels of Fig. 3 confirm the wave identifications by showing EMFISIS search coil data (Kletzing et al. 2013) and by showing (in the fifth panel) the ratio of E/B field values. The ratio E/B indicates the strength of electromagnetic modes for whistler waves and gives clear identification of hiss, magnetosonic, and Alfven wave activity. Clearly, the RBSP sensor provide comprehensive wave measurements throughout the inner magnetosphere.

Such low-energy particle measurements and wave measurements, as shown in Fig. 3, proved indispensable for study of specific radiation belt enhancement events. A notable example of such a powerful radiation belt enhancement event occurred on 17 March 2013. A well-observed solar coronal mass ejection (CME) occurred on 15 March 2013. This CME had associated with it a sharply defined shock wave and this struck the Earth’s magnetosphere at ~ 0604 UT on 17 March (Baker et al. 2014a). The outer radiation belt was rapidly depleted of multi-MeV electrons following the shock impact.

**Fig. 4:**
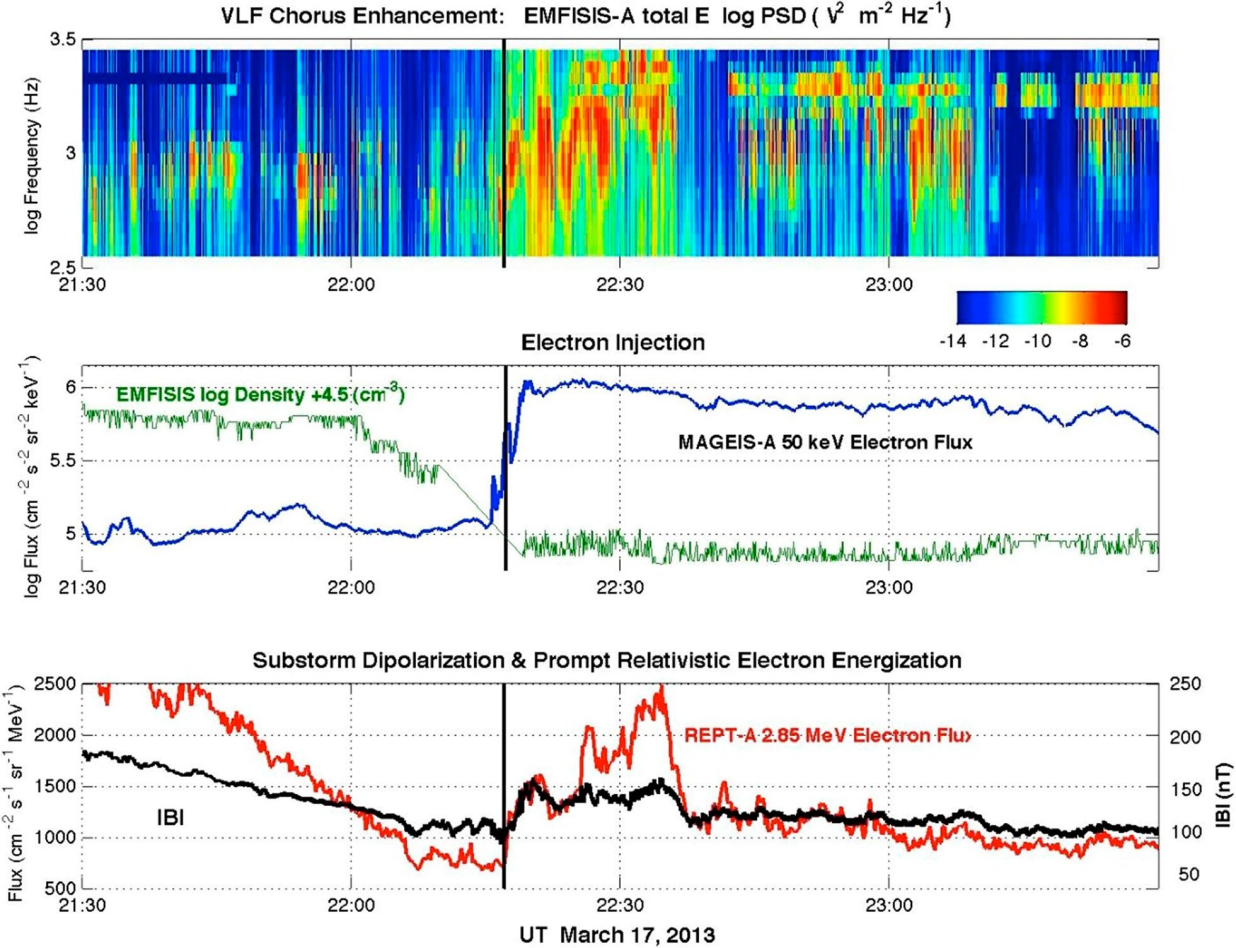
**a** Total electric field power spectral density at VLF chorus band frequencies as observed with the EMFISIS instrument on RBSP-A. **b** Total chorus band wave power between 300 and 3000 Hz and MagEIS 50 keV electron flux (blue curve). **c** EMFISIS magnetic field magnitude (heavy curve) overplotted on REPT-A 2.85 MeV electron flux observations (red curve) ( Adapted from Foster et al. 2014)

As analyzed in detail by Foster et al. (2014), the radiation belt electron population from ~ 1 to ~ 10 MeV remained quite depleted from 0600 UT until ~ 2200 UT on 17 March. Then, at ~ 2200 UT a strong magnetospheric substorm was observed to occur. Figure 4 (from Foster et al. 2014) shows in the middle panel that there was a strong and abrupt energetic (~ 50 keV) electron injection observed by the MagEIS-A sensor (blue trace). Concurrently (green curve), the local plasma density measured by EMFISIS dropped precipitously. These are classic signatures of substorm onset (Baker et al. 1996). The lower panel of Fig. 4 shows also that the magnetic field local to RBSP-A increased rapidly as part of the magnetic field “dipolarization” that characterizes the substorm onset (Baker et al. 1996).

The top panel of Fig. 4 shows that concurrent with the* E* ~ 50 keV electron injection of the substorm, there was an immediate and very distinct enhancement of the VLF chorus waves measured by EMFISIS sensors. Almost immediately (see the lower panel) the *E* = 2.85 MeV electrons measured by the REPT-A sensors began a rapid recovery. As shown by Foster et al. (2014, 2017), substorm events and the associated chorus wave enhancements can rapidly and impressively replenish the entire depleted outer Van Allen radiation zone on a time scale of a few tens of minutes. As further studied by Omura et al. (2019), the nonlinear wave–particle interactions during events such as that on 17 March 2013 through oblique chorus wave interactions can greatly accelerate highly relativistic electrons on time scales of just a few minutes. This demonstrates that Earth’s magnetosphere is a remarkably efficient and effective electron accelerator that uses wave power from storm-generated rising-tone chorus bursts (see Meredith et al. 2003) to accelerate extremely high-energy electron populations in the heart of the outer Van Allen belt.

## Radiation belt structure and dynamics

As remarked upon in the prior section of this paper, strong CME-induced changes of the near-Earth solar wind environment can alter dramatically the large-scale configuration of the radiation belts. As shown by Van Allen Probes studies (Baker et al. 2013b, 2014a, 2016, 2019), solar wind changes caused by CME events can first greatly deplete the vast majority of the outer Van Allen zone electron population and then subsequent geomagnetic storm processes (see, also, Miyoshi et al. 2013) can rapidly replenish the outer belt region (as discussed in prior section). The Van Allen Probes proved to be ideal tools for studying the global-scale morphology changes of the radiation belts from such major solar events over periods of days, weeks, and months. In this section we will examine some of these kinds of broad radiation belt configuration changes.

**Fig. 5:**
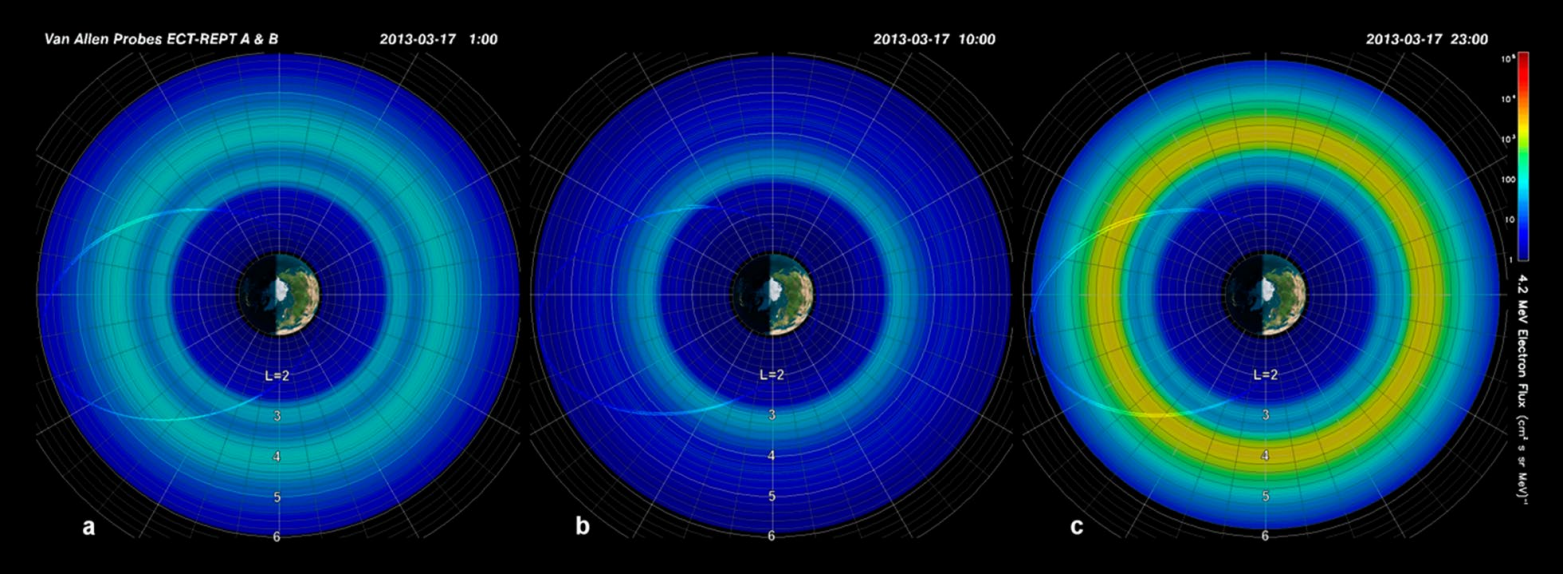
Polar plots of electron fluxes for 4.2-MeV channel for 17 March 2013. **a** Pattern for ~ 0100 UT. **b** For ~ 1000 UT. **c** For ~ 2300 UT for times shown (from REPT = Relativistic Electron–Proton Telescope) (From Baker et al. 2019)

Previously we described the detailed behavior of the outer zone electrons for the specific time that outer radiation belt was depleted (Baker et al. 2014a) and for the time when the outer belt was rapidly repopulated (Foster et al. 2014) during the March 2013 CME-driven storm event. In a subsequent study of the “St. Patrick’s Day” storm of March 2013, Baker et al. (2019) examined the more global features of this same storm event. Here, Fig. 5 shows the globally mapped outer belt configuration for *E* = 4.2 MeV electrons throughout the day on 17 March 2013.

The patterns shown in Fig. 5 are derived using the electron flux measurements along the elliptical orbital trajectories for the RBSP-A and RBSP-B spacecraft. Careful examination of each panel of Fig. 5 shows the trajectory of the RBSP-A spacecraft projected onto the magnetic equatorial plane. The flux of 4.2 MeV electrons at each point along the orbital trajectory is color-coded according to the logarithmic intensity bar to the far right of the figure. Since the electrons measured by the REPT sensors drift azimuthally around the Earth on magnetic drift shells much faster than the spacecraft cuts across *L*-shells, it is sensible (and reasonable) to map the locally measured fluxes all the way around the Earth to indicate the distribution of electrons throughout the entire radiation belt region (Baker et al. 2019).

In Fig. 5a for belt passages early on 17 March (before the IP shock passage at ~ 0600 UT), the outer Van Allen belt had a striking double-peaked structure. The “normal” outer belt stretched from *L* ~ 3.5 out to about *L* = 5.0. However, the electron “storage ring” feature (Baker et al. 2013b) was clearly visible from *L* ~ 2.8 out to *L* ~ 3.2. When one realizes that there also was an inner zone proton belt at the same time (Selesnick et al. 2016), the 17 March pre-storm conditions were a clear example of the three-belt radiation configuration.

In Fig. 5b, the data are shown for the times around 1000 UT on 17 March 2013. This was after the CME-driven shock wave had struck the magnetosphere thereby depleting much of the outer zone. As is evident from the figure, only the storage ring electron population escaped loss and so electrons from *L* ~ 3 to *L* ~ 3.5 remained trapped, while all other outer zone electrons were lost. As described by Baker et al. (2016), many of the electrons at *L* ≳ 4.0 were probably lost outward through the magnetopause, but those closer to the earth (*L* ≲ 4.0) were probably scattered by whistler waves into the atmospheric loss cone.

Figure 5c shows *E* = 4.2 MeV data for the period late on the day of 17 March 2013. This is after the strong substorm activity studied by Foster et al. (2014). By this time, the outer Van Allen belt was broadly replenished and much enhanced over the pre-storm electron intensities. The outer zone was wider in radial extent than prior to the shock impact and the outer belt was orders of magnitude higher in absolute fluxes. However, note that the storage ring (third belt) was about the same in position and strength throughout this event period.

**Fig. 6:**
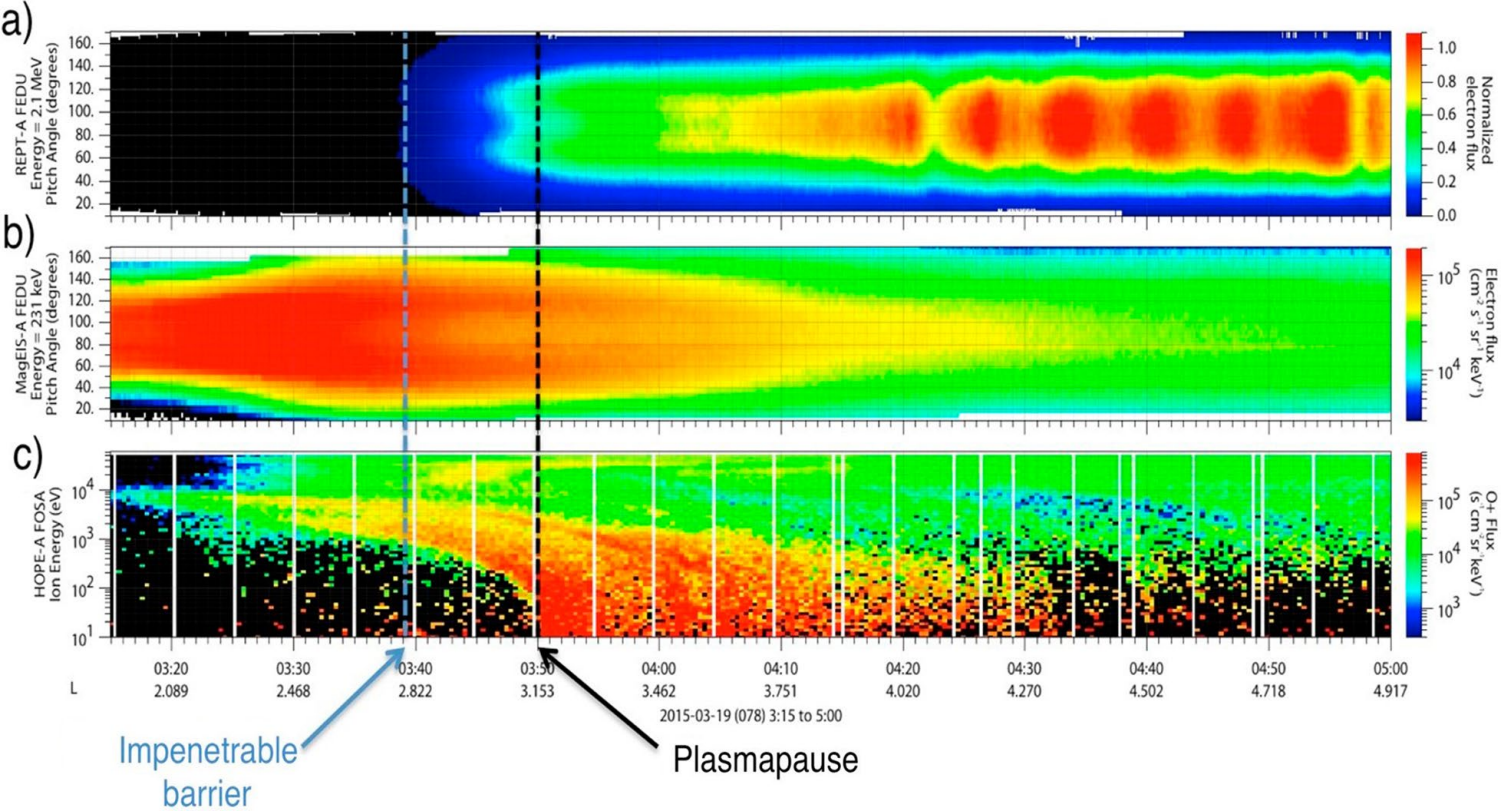
Van Allen Probe-A data for 19 March 2015, 0315–0500 UT. **a** Pitch angle distributions for 2.1 MeV electrons from 1.5 ≲ *L* ≲ 5. **b** Pitch angle distributions for 231 keV electrons. **c** Spin-averaged low-energy oxygen energy spectrogram (From Baker et al. 2016)

An event having many similarities to the March 2013 storm period occurred almost exactly 2 years later in March 2015. This 2015 St. Patrick’s Day event was compared and contrasted with the 2013 event in the papers by Baker et al. (2016,  2019). Figure 6 shows several types of Van Allen Probes (RBSP-A) data for the period of 0315 UT to 0500 UT on 19 March 2015. The upper panel shows REPT-A electron data for the *E* = 2.1 MeV channel. The data are shown as color-coded pitch-angle resolved electron intensities versus time. The second panel (Fig. 6b) shows electron data for the *E* = 231 keV MagEIS channel in a format similar to that in Fig. 6a. Finally, Fig. 6c (lower panel) shows the energy-time spectrogram (also color-coded) for the RBSP-A HOPE oxygen channels.

Marked on the Fig. 6 panels by the black vertical dashed lines is an indication of the plasmapause location (inferred from the concurrent EFW and EMFISIS wave sensors). Also shown by the blue vertical dashed line is the innermost extent of the highly relativistic electrons as measured by REPT-A. (This is termed the “Impenetrable Barrier” and will be discussed further below). As is clear in Fig. 6a, no discernable flux of *E* = 2.1 MeV electrons was observed inside of *L* ~ 2.8 on this post-storm day. Near and just inside the plasmapause, these electrons had a clear “butterfly” (bimodally peaked) pitch angle distribution. As described by Baker et al. (2016), this double-peaked distribution may have been due to local acceleration associated with magnetosonic waves interacting with lower energy (hundreds of keV electrons) near the plasmapause (reference Fig. 3).

Note in Fig. 6 that no multi-MeV electrons were seen to move inside of *L* = 2.8. This same fact was true in the March 2013 event period (see Fig. 5). However, the hundreds of keV electrons measured by MagEIS (Fig. 6b) were able to penetrate freely inward to *L* ≲ 3 and seemed unfazed by the plasmapause boundary. In addition, as shown in Fig. 6c, energetic oxygen (O^+^) ions were able to penetrate deeply into the inner magnetosphere in considerable contrast to the high-energy electrons. After $\sim$ 0400 UT on this day—and in the outer part of the outer Van Allen zone—there were high fluxes of *E* = 2.1 MeV electrons (*L* ≳ 3.5) showing strongly trapped (90° pitch angle) electrons.

The March 2015 storm period produced some of the strongest relativistic electron enhancements during the entire Van Allen Probes era (see Baker et al. 2016, 2019). As such there were strong enhancements from *L *~ 2.8 throughout the outer magnetosphere out to (and beyond) the geostationary orbit (*L* ~ 6.6). Fluxes of electrons increased significantly up to energies above 10 MeV during the main storm period of 17–20 March 2015 (see Baker et al. 2019).

**Fig. 7:**
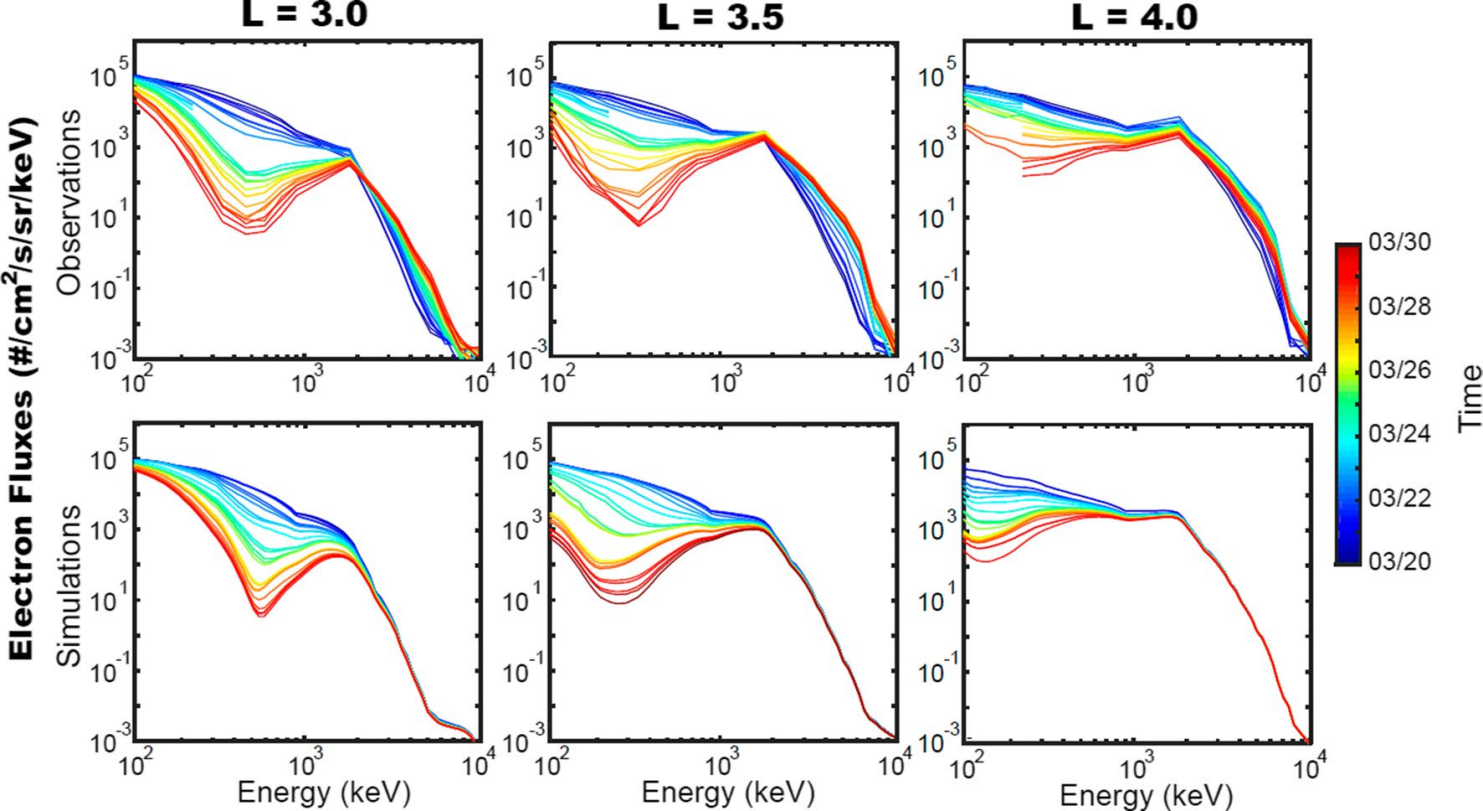
Comparison of Fokker–Planck simulations (bottom row of three panels) with observations of energetic electron spectra made in March 2015 by the RBSP spacecraft. The color coding indicates the dates when data were acquired from 20 to 30 March 2015 ( Adapted from Zhao et al. 2019a)

Following the storm interval itself, the energetic electrons near the heart of the outer radiation zone showed fascinating evolution of the overall electron energy spectrum. This was studied in detail by Zhao et al. (2019a). Figure 7 shows combined data from the MagEIS and REPT sensors for the period 20 March to 30 March. The upper row of panels show energy spectra for electrons from 100 keV to 10 MeV. The left most panel is for *L* = 3.0. The middle column of Fig. 7 is for *L* = 3.5 and the right column is for *L* = 4.0. The color-coding shows the data of each energy spectrum with deep blue corresponding to 20 March all the way up to bright red corresponding to spectra taken on 30 March.

As described in the study of Zhao et al. (2019a) the overall electron energy spectra acquired around 20 March were a “broken” power law with a hard spectrum from 100 keV to about *E* = 2 MeV and then a steeper spectrum from 2 MeV up to 10 MeV. This spectral shape applied across the range from *L* = 3 or to beyond *L* = 4. However, as time progressed from 20 March forward, the fluxes of electrons from ~ 200 keV to ~ 1 MeV began to diminish substantially. By 30 March (as shown by the reddish spectral traces) there was a dramatic diminution of medium energy electrons even though the electron fluxes above *E* ~ 1 MeV were hardly changed. This led to what Zhao et al. termed a “reversed” energy spectrum.

Using observed wave data from the EMFISIS sensors onboard the RBSP spacecraft, Zhao et al. modeled the interaction of hiss waves with the initial (20 March) electron energy spectrum. The lower row of panels in Fig. 7 show the modeled evolution of the energy spectra at the several L positions as a function of time. Clearly, these results show that hiss waves acting to scatter (and thus precipitate) hundreds of keV electrons explains almost perfectly the spectra seen for energetic electrons after the 2015 St. Patrick’s Day storm. Also quite clearly, the hiss waves were expected to have little effect on the multi-MeV electron population.

## Anthropogenic effects on the radiation belts

One of the most striking and persistent morphological features seen in the multi-MeV data from the Van Allen Probes was the sharp inner extent of the outer radiation belt. This feature is evident in Figs. 5 and 6 shown above and was the precise focus of a dedicated paper by Baker et al. (2014b). As commented previously in this review, Baker et al. (2014b) termed the sharp inner edge of the outer Van Allen belt as the “impenetrable barrier”, because even during strong solar wind forcing during the RBSP era, multi-MeV electrons were never seen to move inward of an equatorial radial distance of *r* ~ 2.8 *R*_E_. This *L* ~ 2.8 (± 0.1) inward extent of the relativistic electrons seemed to persist, often for weeks or months at a time.

In the original study, Baker et al. (2014b) attributed the “barrier” to natural effects, where increasing geomagnetic field strengths and diminishing pitch angle diffusion would slow any inward transport of very energetic electrons. In this picture, it simply was a natural consequence of greatly slowed inward radial diffusion that the barrier always appeared right around *L* = 2.8.

**Fig. 8:**
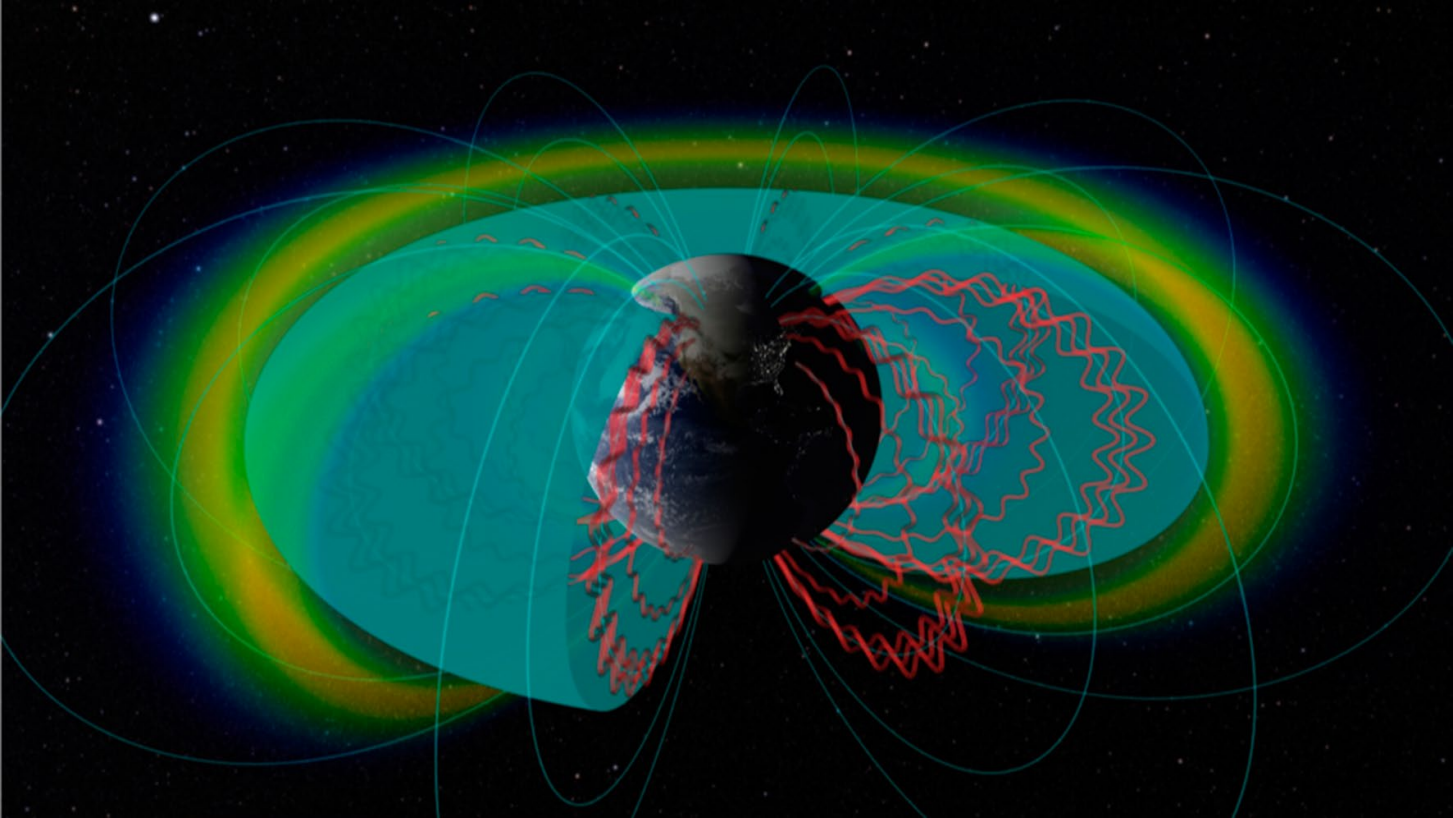
Schematic diagram illustrating the inner part of Earth’s magnetosphere. The outer edge of the VLF “bubble” is sketched showing intense waves confined inside this boundary (Courtesy of NASA)

In subsequent studies, however, the REPT team studied the impenetrable barrier from a different perspective (Foster et al. 2016, 2020). In this later work, it was recognized that plasma waves tended to be ducted along magnetic field lines (especially inside the plasmasphere) and can represent the presence of quite high wave power relatively close to the Earth. Figure 8 illustrates notionally that waves triggered by mid-latitude lightning or from powerful human transmitters can populate a Very Low Frequency (VLF) “bubble” (Foster et al. 2016).

**Fig. 9:**
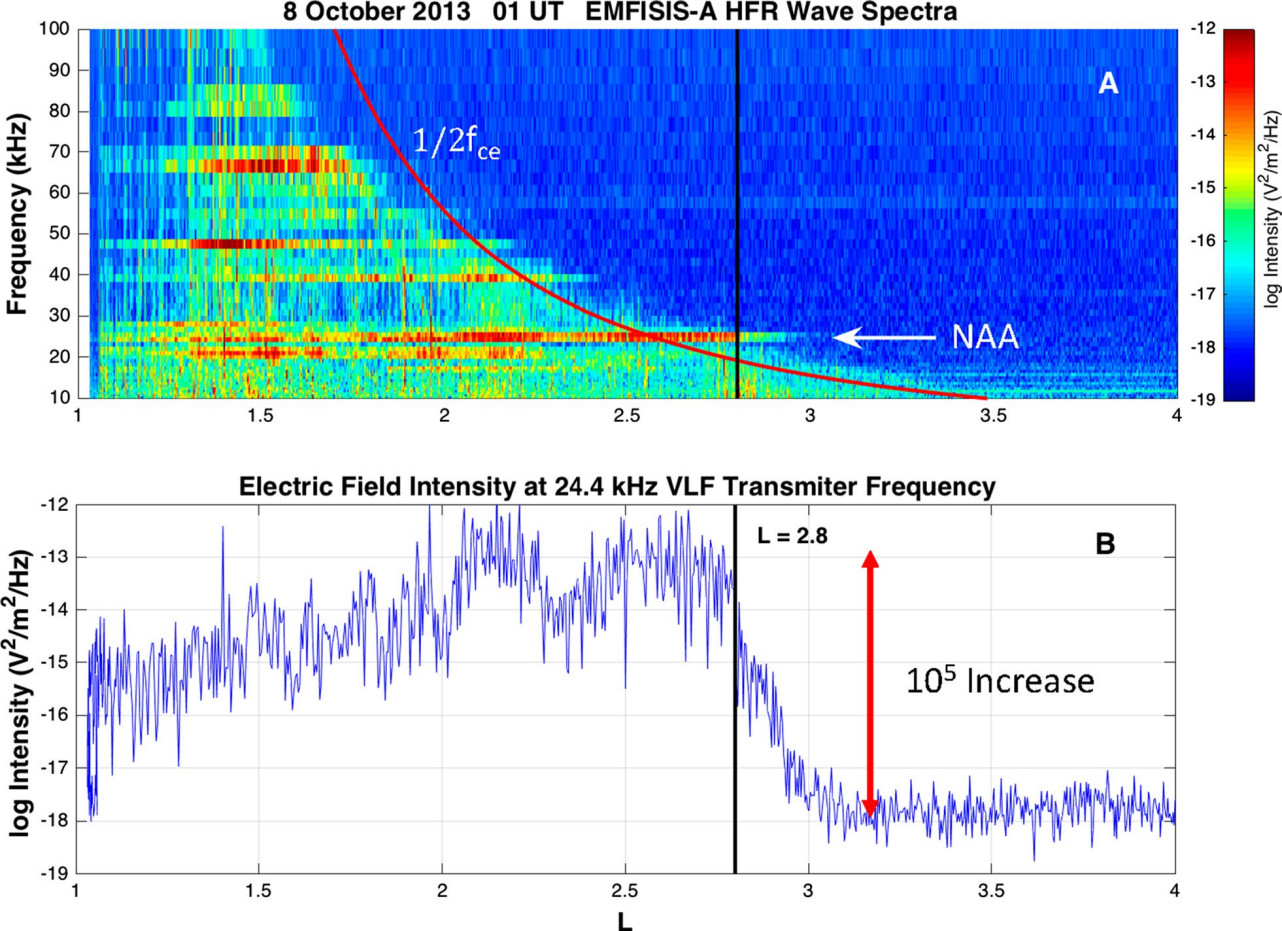
EMFISIS observations reveal the confinement of the signals from ground-based VLF transmitters to a magnetically confined bubble surrounding Earth. **a** Spectra of wave electric field intensity are plotted versus L for a perigee pass in October 2013 at a time when the plasmapause was beyond *L* = 4. The red curve denotes 0.5 fce. An arrow denotes the strong VLF transmitter signal near 24 kHz. **b** Electric field intensity (|*E*|^2) at the transmitter frequency increased by 10^5 over 0.5 RE as Van Allen Probe A moved inward across the outer edge of the VLF bubble (From Foster et al. 2016)

The Van Allen Probes sensors proved to be very useful for studying the VLF bubble properties. Figure 9 shows EMFISIS high-frequency receiver data for a selected day (8 October 2013). The data presented in the upper panel show a frequency vs. *L* value color spectrogram for the HFR instrument. The smooth downward sweeping red curve in Fig. 9a is the value of one-half of the electron gyrofrequency (½ fce) based on the magnetic field strength across the shown *L* value range. The color-coded data generally lying below and to the left of the ½ fce trace are the wave powers detected by the HFR sensor. As is clear from the color bar to the right of the panel, there was mid-level broadband power across most of the frequency range below ½ fce. However, there were also very intense power levels at discrete frequencies ranging from ~ 20 kHz on up to ~ 65 kHz. As noted by Foster et al (2016), the discrete frequencies between 20 and 30 kHz correspond to powerful human transmitters (ground-based) for communication with navy submarines.

The lower panel of Fig. 9 shows the integrated power at the frequency f = 24.4 kHz which is the operating frequency of the U.S. naval transmitter at Cutler, Maine. The integral power is plotted at each radial distance (*L* value) from *L* = 1.0 to *L* = 4.0. As is clear from Fig. 9b, the VLF “bubble” extends out to *L* = 2.8. The integrated power at 24.4 kHz falls off by a factor of 10^5^ right at the location of the impenetrable barrier identified in the REPT data.

In their work, Foster et al. (2016, 2020) have examined the ways in which the VLF waves inside the “bubble” could act to scatter or inhibit the local acceleration of high-energy (E ≳ 1 MeV) electrons, thereby greatly inhibiting such electrons from diffusing closer to the Earth than *L* ~ 2.8. While more work needs to be done to reach full understanding (including the relative importance of multiple processes acting in this region), it seems clear that human radio transmitters are playing a role in the remarkable, sharply delineated inner edge of the outer Van Allen belt (Foster et al. 2020).

## Summary

Many prior published papers based on the Van Allen Probes particle and field data (including statistical studies; see Gu et al. 2020) have examined various aspects of radiation belt electron acceleration and loss processes. The goal of this present brief review paper has been to consider how certain wave modes in the magnetosphere play a key role in determining the overall structure and time variability of energetic electrons, especially those in the highly relativistic and ultra-relativistic energy domains.

**Fig. 10:**
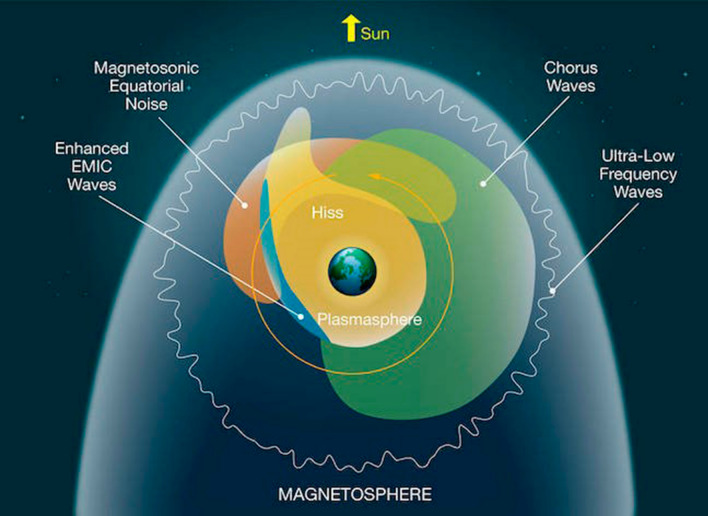
Schematic diagram showing many of the wave modes that affect energetic electrons in the Earth’s radiation belts (courtesy of NASA; adapted from Summers et al. 1998)

Figure 10 is an adaptation of the classic diagram of Summers et al. (1998) that portrays the spatial locations of many key waves that affect the radiation belt properties. In this view—looking down onto the magnetic equatorial plane from above the Earth’s north pole—one sees regions, where different wave populations are prevalent. As we have described briefly in this paper, ultra-low-frequency waves in the outer portions of the radiation belts can produce clear flux modulations and acceleration (under many conditions) as has been known for a long time (Rostoker et al. 1998). As we have noted here, ULF waves initiated by shock impacts on the dayside magnetosphere can produce rather dramatic and long-lasting effects on the relativistic electron populations at (and near) geostationary orbit altitude (see Fig. 1).

A dominant wave mode affecting radiation belt electrons are the chorus waves that are prevalent outside the plasmasphere, especially in the post-midnight and local morning sectors. As we have described here, lower band chorus (i.e., waves a frequencies below ½ fce) have been shown time and again to be remarkably effective at accelerating electrons throughout the outer Van Allen zone to hundreds or thousands of keV energies. The Van Allen Probes made major progress in detailing this chorus wave acceleration story including the role of magnetospheric substorms to provide seed particles and to amplify chorus waves.

As shown in Fig. 10, an important wave mode bridging the local noon sector is the magnetosonic wave population. Such waves outside the plasmapause produce acceleration of electron around *E* = 1 MeV and generate interesting pitch angle distributions (such as those show here in Fig. 6a). The combination of lower-band chorus and magnetosonic waves can explain a good deal of the energy distributions and pitch angle properties that have been documented in Van Allen Probes statistical studies (e.g., Zhao et al. 2019b).

In the limited space available here, we were not able to address the clear effects of electromagnetic ion cyclotron (EMIC) waves on relativistic electrons deep inside the outer Van Allen zone. However, as shown by Usanova et al. (2014), Nakamura et al. (2019), EMIC waves can be shown time and again to strongly scatter off-equatorial high-energy electrons (i.e., α ≠ 90°) causing rapid and effective loss of trapped electrons into the atmosphere.

Finally, plasmaspheric hiss plays a key role in the loss of hundreds of keV electrons in the outer zone. As reviewed here, the work of Zhao et al. (2019a) showed the previously unknown feature of a reversed relativistic electron energy spectra due to hiss interactions following geomagnetic storm events. This work has proven both qualitatively and quantitatively compelling to explain new features in the high-energy electron picture for the radiation belts (see Fig. 7).

As a last point, we note that the diagram in Fig. 10 does not explicitly illustrate the VLF wave populations near Earth that we described here as the VLF “bubble” (see Figs. 8 and 9). As we have reviewed here the probable anthropogenic effects of human radio transmitters (ground-based) also need very much to be fit into the pantheon of natural wave phenomena to round out the remarkable wave–particle interaction story that the Van Allen Probes were able to tell.

## Data Availability

Data are available from the RBSP Gateway at JHU/APL.
